# Motion database of disguised and non-disguised team handball penalty throws by novice and expert performers

**DOI:** 10.1016/j.dib.2017.10.042

**Published:** 2017-10-27

**Authors:** Fabian Helm, Nikolaus F. Troje, Jörn Munzert

**Affiliations:** a Department of Psychology and Sport Sciences, Goethe-University Frankfurt, Germany; b Department of Psychology and Sport Sciences, Justus-Liebig-University Giessen, Germany; c Department of Psychology, Queen's University, ON, Canada

**Keywords:** Motion capture data, Disguise, Expertise

## Abstract

This article describes the motion database for a large sample (*n* = 2400) of 7-m penalty throws in team handball that includes 1600 disguised throws. Throws were performed by both novice (*n* = 5) and expert (*n* = 5) penalty takers. The article reports the methods and materials used to capture the motion data. The database itself is accessible for download via JLU Web Server and provides all raw files in a three-dimensional motion data format (.c3d). Additional information is given on the marker placement of the penalty taker, goalkeeper, and ball together with details on the skill level and/or playing history of the expert group. The database was first used by Helm et al. (2017) [1] to investigate the kinematic patterns of disguised movements. Results of this analysis are reported and discussed in their article “Kinematic patterns underlying disguised movements: Spatial and temporal dissimilarity compared to genuine movement patterns” (doi:10.1016/j.humov.2017.05.010) [1].

**Specifications Table**

**Table:** 

Subject area	*Human movement science*
More specific subject area	*Movement analysis of sports performance*
Type of data	*Motion capture data*
How data was acquired	*VICON Motion Capture System (Vicon Motion Systems Ltd., Oxford, UK)*
Data format	*Raw 3D motion data (.c3d-files), sampled at 240 Hz*
Experimental factors	*Expertise, Type of throws*
Experimental features	*Ten right-handed expert (n = 5) and novice (n = 5) penalty takers conducted a total of 2400 7-m team handball penalties in two conditions (disguised, non-disguised) with further different variations that included 1600 disguised throws (see*Table 1*for more details).*
Data source location	*Giessen, Germany*
Data accessibility	*Data accessible via JLU Web Server* (*MotionDatabase.zip*)

**Value of the data**

• Large database for analyzing and/or synthesizing the kinematic patterns linked to disguised movements.
• Particular relevance to the fields of human movement sciences and/or perceptual (anticipation) research.
• In their raw data format also relevant for research on developing methods to preprocess motion capture data.

## 1 Data

This brief article describes and provides the raw motion data of 2400 7-m handball penalties including 1600 disguised throws performed by novice (*n* = 5) and expert (*n* = 5) penalty takers. The data were recorded by means of a VICON motion capture system (Vicon Motion Systems Ltd., Oxford, UK) equipped with 15 CCD high speed cameras. The system tracked three-dimensional (Cartesian) coordinates of retroreflective markers with a spatial accuracy of 1 mm and a sampling rate of 240 Hz. The reconstruction of marker locations was computed in Nexus 1.8.5 (Vicon Motion Systems Ltd., Oxford, UK). Table 1 explains the structure of the data set on the basis of the specifications of the experimental conditions. The data are accessible for download via JLU Web Server (*MotionDatabase.zip*). Originally, these data were collected for the analysis and synthesis of disguised movement patterns described in Helm et al. [1].

**Table 1:** *Specification of Experimental Conditions.* The trial structure of the database is taken from the arrangement of penalty throws according to the experimental conditions.

**Condition**	**#**	**Target location**			**Description**
**No Disguise**	1	Upper Right			Straight throw to the target at the specified target location.
	2	Upper Left			
	3	Lower Right			
	4	Lower Left			
**Disguise**		*First action*	–	*Second actions*	
	5	Upper Right	–	Upper Left	Disguised throw (first action) to the first target location, followed by a straight veridical throw (second action) to the then specified target location.
	6	Upper Left	–	Upper Right	
	7	Lower Right	–	Lower Left	
	8	Lower Left	–	Lower Right	
	9	Upper Right	–	Upper Right	
	10	Upper Left	–	Upper Left	
	11	Lower Right	–	Lower Right	
	12	Lower Left	–	Lower Left	

## 2 Experimental design, materials, and methods

### 2.1 Participants

Ten right-handed male participants volunteered to participate in this study (mean age = 22.1, *SD* = 3.5 years). They were divided into two different expertise groups: experts (competitive elite team handball field players, according to Swann et al., [2] *n* = 5) and novices with no other previous experience in team handball other than attending a university class for beginners (*n* = 5). Participants from the expert group played in one of the four highest national leagues in Germany and were frequent penalty takers for their team. More details on participants’ playing history can be found in Table 2.

**Table 2:** *Personal Details of Expert Penalty Taker Group.* Personal details (age, weight, height) including participants’ team handball experience are described separately for each individual member of the expert group.

Participant	Age	Weight (kg)	Height (cm)	Club Level in GER	Years of Practice	Hrs/Week
SUB_E01	20	78	185	2nd League	15	12
SUB_E02	17	66	179	4th League	12	12
SUB_E03	22	95	186	2nd League	18	13
SUB_E04	24	78	190	3rd League	16	6.5
SUB_E05	21	86	185	1st/3rd League	15	16
					*M* = 15 (SD = 2.2)	13 (1.9)

### 2.2 Materials and experimental design

**Fig. 1:**
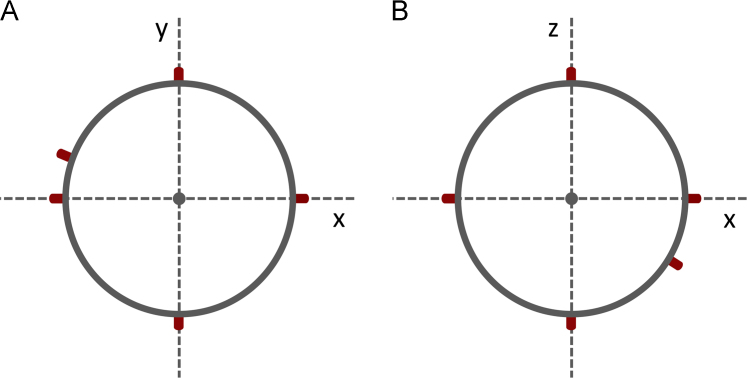
*Marker Placement of Ball*. Locations of the marker placement on the ball surface are described for the xy- (A) and xz-plane (B). Six markers are placed symmetrically on the straight lines defined by the ball's diameter, whereas two markers are placed asymmetrically so that the marker positions can be determined distinctively in three-dimensional space.

Four different target locations (1.2 m × 1.2 m in size) were set up in the upper and lower left and right corners of a standard handball goal (3 m × 2 m, as specified in the guidelines of the International Handball Federation). Kinematic data of the penalty takers, the goalkeepers, and the ball were recorded with the above-mentioned motion capture system. A standard full-body 41-marker placement was used for the penalty takers, whereas only 16 markers were attached to the goalkeepers. The goalkeepers’ minimized marker set can be used only to define the onsets and target directions of the defensive reactions. Eight markers were placed on the ball. A detailed description of all marker placements is given in Table 3, Table 4 and in Fig. 1. Participants wore tight neoprene shirts and shorts, but most markers were attached directly to the skin. Markers on the head and wrists were attached to elastic bands, and those on the feet (if applicable) were taped to the subjects’ shoes.

**Table 3:** *Marker Placement of Penalty Takers.* Labels and locations of the 41 body markers attached to the penalty takers are described according to the specifications given for standard full-body marker placement by Vicon Motion Systems, Oxford, UK. Most markers are placed symmetrically on locations of the left and right half of the body. Exceptions are some markers on the arm and leg (e.g., upper-/forearm markers and thigh/shank markers).

**Penalty Taker: Standard full body 41-marker placement**	
**Head Markers**	
Left front head	Located approx. over the left temple.
Right front head	Located approx. over the right temple.
Left back head	Placed on the back of the head, roughly in a horizontal plane of the front head markers.
Right back head	
**Torso Markers**	
7th cervical vertebrae	Spinal process of the 7th cervical vertebrae.
10th Thoracic vertebrae	Spinal process of the 10th thoracic vertebrae.
Clavicle	Jugular notch where the clavicles meet the sternum.
Sternum	Xiphoid process of the sternum.
Right back	Placed in the middle of the right scapula.
**Arm and Hand Markers (Left and Right)**	
Shoulder	Placed on the acromioclavicular joint.
Upper arm marker	Placed on the upper arm btw. elbow and shoulder markers. Upper arm markers are asymmetrically placed for left and right.
Elbow	Placed on lateral epicondyle approximating elbow joint axis.
Forearm	Placed on the lower arm btw. wrist and elbow markers. Left and right forearm marker are placed asymmetrical.
Wrist A	Lateral (thumb) side of the wrist.
Wrist B	Medial (pinky) side of the wrist.
Hand	Placed on the dorsum of the hand just below the head of the second metacarpal.
**Pelvis Markers**	
Left anterior superior iliac spine (ASIS)	Placed directly over the anterior superior iliac spine.
Right ASIS	
Left posterior superior iliac spine (PSIS)	Placed directly over the posterior superior iliac spine.
Right PSIS	
**Leg and Foot Markers (Left and Right)**	
Thigh	Placed over the lower lateral 1/3 surface of the thigh. Left and right thigh marker are placed asymmetrical.
Knee	Placed on the lateral epicondyle of the knee.
Tibial wand	Similar to thigh marker. Placed over the lower 1/3 of the shank. Left and right tibial wand marker are placed asymmetrical.
Toe	Placed over the first metatarsal head, on the mid-foot side of the equinus break btw. fore- and mid-foot.
Little Toe	Placed over the fifth metatarsal head.
Heel	Placed on the clacaneous at same height above the plantar surface of the foot as toe marker.

**Table 4:** *Marker Placement of Goalkeepers.* This table reports the labels and locations of the minimized 16-marker placement on the goalkeeper. Markers on the left and right half of the body are placed asymmetrically except for those attached to the hand and thigh.

**Goalkeeper: Individual 16-marker placement**	
**Head Markers**	
Left front head	Located approx. over the left temple.
Right front head	Located approx. over the right temple.
Left back head	Placed on the back of the head, roughly in a horizontal plane of the front head markers.
Right back head	
**Upper and Lower Body Markers (Left and Right)**	
Wrist A	Lateral (thumb) side of the wrist.
Wrist B	Medial (pinky) side of the wrist.
Hand	Placed on the dorsum of the hand below the head of the second metacarpal. Left and right hand markers are placed asymmetrical.
ASIS	Placed directly over the ASIS.
Ankle	Placed on the lateral malleolus along an imaginary line that passes through the transmalleolor axis.
Thigh	Placed asymmetrically (left vs. right) over the lower lateral 1/2 surface of the thigh.

In general, an in-situational experiment with two expertise groups (novices vs. experts) in two different conditions (disguise, no disguise) was conducted. In line with the in-situational requirements, goalkeepers with the same skill level as the penalty takers volunteered to participate actively in the study to make the situation as realistic as possible.

### 2.3 Throwing procedure

Each participant conducted a total of 240 7-m penalty throws in two conditions with different variations including 160 disguised throws (see Table 1 for more details). When performing the disguised throws, the penalty takers tried to mimic a genuine throw without final ball release (disguised part) toward a specific target location, and then immediately continued the action with a repetition of the throwing movement resulting in a final ball release. The disguised actions were characterized by a deliberate attempt to mask the actual intent to “abort” the first throw. Instructions (type of throw: disguise or no disguise; and target location) were given on a screen in pseudo-randomized order via Presentation software (Neurobehavioral Systems, Albany, NY, USA).
